# Resolution of a Greater Than 50-year History of Severe, Chronic Low Back Pain Following an Ultrasound-guided Platelet-rich Plasma Infiltration of the Thoracolumbar Fascia

**DOI:** 10.7759/cureus.3457

**Published:** 2018-10-16

**Authors:** Andre Panagos

**Affiliations:** 1 Physical Medicine and Rehabilitation, New York University / Langone Medical Center, New York, USA

**Keywords:** fascia, low back pain, chronic back pain, platelet-rich plasma, ultrasound, musculoskeletal ultrasound, musculoskeletal symptoms, lumbar spine, lumbar pain, pain management

## Abstract

The diagnosis of chronic low back pain is a scourge of society that does not take into account the pathoanatomical cause of pain. This case describes a six-year search for the pinpoint pathoanatomical diagnosis of a patient’s 50-plus year history of debilitating chronic low back pain after he failed the standard nonoperative and operative treatment modalities. Ultrasound-guided diagnostic blocks identified a potential space within the thoracolumbar fascia. This was treated with platelet-rich plasma, yielding a complete resolution of his pain and a full return to normal activities of daily living for three years since the procedure.

## Introduction

Low back pain is one of the most challenging conditions to treat, as it is a symptom of an underlying disorder. Low back pain is incredibly frustrating for clinicians to treat, as over 100 conditions can result in back pain [1]. It is one of the most prevalent musculoskeletal disorders in developed countries, affecting up to 85% of the adult chronic pain population. Also, a precise pathoanatomical diagnosis cannot be determined in up to 85% of patients with low back pain [2], so treatment is based on the classic step-wise approach. For those unfortunate patients who do not respond, chronic pain management is advised to mitigate the effects of the pain on patient function with an attempt to approximate as close to a normal lifestyle as possible. Recently developed technologies have opened up new avenues for diagnosis and treatment. In this setting, musculoskeletal sonography has become an invaluable tool, which helped to determine the source of pain in this patient at the thoracolumbar fascia and to guide the definitive treatment using platelet-rich plasma.

## Case presentation

A 65-year-old, deconditioned male was referred for the evaluation and treatment of chronic low back pain. He reported a history of back pain that began in 1960 when he was hit in the lower back during a high school football game. Imaging noted fractures of the right L4 and L5 transverse processes and an L4-5 disc herniation, which was treated with rest. He was injured again during a pick-up rugby game in 1963, which resulted in the use of a rigid Boston brace for three months to treat L4-5 instability. He developed increasing episodes where he lost the ability to weight bear on his right lower extremity, resulting in a non-instrumented L4-S1 lumbar fusion in 1972.

He unsuccessfully trialed various pain medications, such as hydromorphone, morphine, oxycodone/acetaminophen, tramadol, gabapentin, lidocaine patches, and capsaicin patches, over many years. He eventually developed severe opioid-induced pruritus, which was treated with diphenhydramine. If the pain was severe, he would take a hydromorphone. He had many prior lumbar epidural steroid injections and local ketorolac and lidocaine injections, sometimes weekly, in the emergency room.

I met the patient in 2009 when he transferred his care to our office. His physical examination was most pertinent for visual analog scale (VAS) 1-10/10 pain in the right buttock that radiated into the right leg with associated numbness and tingling in the right foot. On examination, his strength was 4/5 in the right hip flexors and 5/5 in the remaining lower extremity muscles with intact ankle reflexes bilaterally. His sensation was also intact to light touch throughout the lower extremity dermatomes. Bilateral straight leg raise signs were negative. On palpation, he had very tight bilateral lumbar paraspinal muscles that were diffusely sensitive. His pain was often severe, and on multiple visits to our office, he would very slowly shuffle into the office using a single-point cane under a great deal of distress.

Subsequent lumbar X-rays in 2010 demonstrated no abnormal lumbar motion with flexion and extension and pelvic and right hip X-rays in 2011 were unremarkable. We could not order a magnetic resonance imaging (MRI) study due to his implantable pacemaker. His most recent lumbar computed tomography (CT) scan in 2010 noted an osseous fusion of the facet joints from L4-S1, a mild disc bulge at L5-S1, moderate sacroiliac joint degeneration, and mild T12 and L1 anterior wedging, with no change compared with the 2007 study.

**Figure 1:**
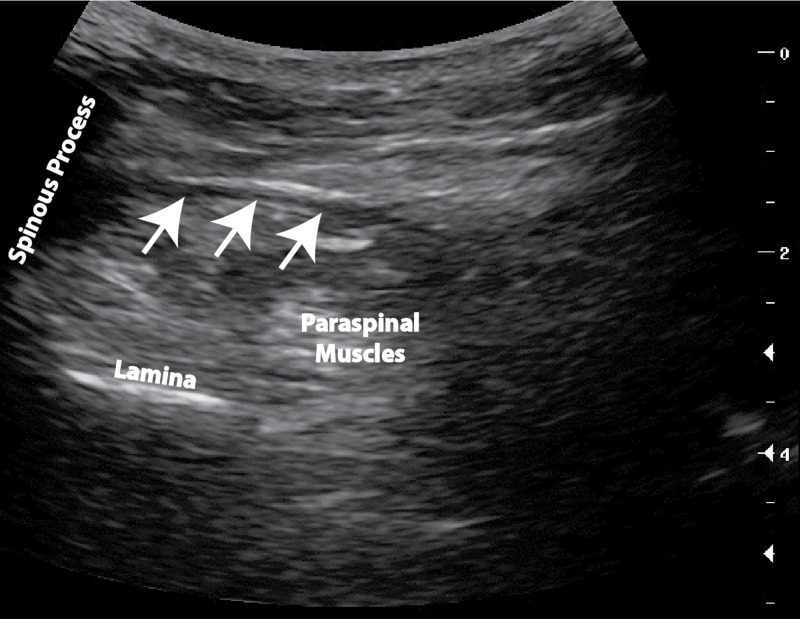
Ultrasound image of the fascial tear This is a cross-sectional ultrasound image of the right paraspinal muscles at the L5-S1 vertebral body level. The arrows outline the fascial separation to the right of the L5 spinous process that was targeted and demonstrated laminar flow into a potential space.

He was not interested in chronic opioid or adjuvant therapy other than for occasional hydromorphone with diphenhydramine for severe exacerbations. We trialed physical therapy, which always exacerbated his pain. In total, during our attempt to manage his pain, he had 10 right L5-S1 epidural steroid injections as well as a caudal epidural injection and over 20 blind and ultrasound-guided right L5-S1 paraspinal trigger point injections with corticosteroids and anesthetic. These procedures only improved his pain for several days, but they were effective enough to allow him to walk normally out of the office after shuffling in. An ultrasound-guided right hip joint corticosteroid injection in 2011 was not helpful. During one of these trigger point injections on January 29, 2014, we noticed a laminar flow of the triamcinolone and 1% lidocaine injectate within the thoracolumbar fascial plane (Figure 1). Several days later, he reported an uncharacteristically remarkable improvement, yet it was again temporary.

Although the thoracolumbar fascia is connective tissue that separates the individual erector spinae muscles, an anatomical cavity is not known to exist in this region. On February 6, 2014, we proceeded with an ultrasound-guided right L5-S1 thoracolumbar fascia plane platelet-rich plasma infiltration using a total volume of 8 ml. By March 11, 2014, the patient reported a significant improvement in his pain with eventual resolution. He reported that he could accomplish all activities without pain, including walking unlimited distances and running for the bus with no discomfort. Over time, he also began to engage in heavy yard work, which he had not done for decades and was no longer spending weeks lying in bed. This improvement continued through the last follow-up in 2017.

## Discussion

This case presentation highlights a new etiology for chronic low back pain that only recently has been possible to diagnose and treat due to advances in musculoskeletal sonography and regenerative medicine, respectively. In this case, traditional imaging techniques and treatment modalities provided temporary relief but failed to resolve the patient’s chronic back pain. A review of the English literature found in PubMed did not yield any articles that documented full resolution of low back pain due to a tear of the thoracolumbar fascia decades after the original injury.

Although this injury has been dynamic over the past 50-plus years, it was most likely due to the original blunt trauma and resulting L4-5 instability. We trialed trigger point injections and epidural injections with only moderate improvement, which ruled out an intrinsic muscle injury/spasm and a radiculopathy, respectively, as a source of pain. In research assessing delayed onset muscle soreness (DOMS), an injection of hypertonic saline within the fascia was found to result in more pain than a similar injection into the muscle itself [3] due to the sensitized free nerve endings and encapsulated mechanoreceptors within the thoracolumbar fascia [4]. The patient had tremendous improvement after the corticosteroid injection within the thoracolumbar fascial plane, which suggested that we had found the original pain generator.

The thoracolumbar fascia is thought to act as an internal ectoskeletal corset that transmits force between the arms and legs while protecting the static and contractile elements within the spine by minimizing friction [5]. Long-lasting sensitization to mechanical and chemical stimulation in the deeper tissues has been found to occur if nerve growth factor is injected into the erector spinae [6]. Some researchers also believe that the progressive in-growth of nociceptive fibers into the thoracolumbar fascia can occur as has been documented within the fascia in patellofemoral malalignment [7]. The primary sensory fibers found are peptidergic calcitonin gene-related peptide (CGRP) and substance P-containing fibers. They are known to cause neurogenic inflammation that can result in local vasodilation and increased blood vessel permeability. They can also invade the nerve endings antidromically [8].

Platelet-rich plasma is thought to work through the release of growth factors in areas of tissue damage. The alpha-granules in platelets contain many growth factors that are responsible for the initiation and maintenance of the healing response. The growth factors that are released include platelet-derived growth factor (PDGF), transforming growth factor beta (TGF-beta), vascular endothelial growth factor (VEGF), and fibroblast growth factor (FGF). The platelets begin secreting their proteins within 10 minutes of clotting with more than 95% of the growth factors secreted within one hour. Platelets continue to secrete additional proteins for the remainder of their five to 10-day lifespan. The fibrin matrix that forms also has an additional stimulatory effect on healing by trapping platelets and providing an initial matrix for fibroblast migration [9].

Central sensitization is the amplification of neural signaling within the central nervous system that causes pain hypersensitivity not only at the site of pain but in the spinal cord and brain as well [10]. It is thought to be the primary reason chronic back pain is virtually impossible to treat. It is possible that there is bi-directional neurological input that is responsible for the development and maintenance of central sensitization. In this case, it is possible that nociceptive input in the periphery resulted in the development of central sensitization. Once this nociceptive input was removed, the phenomenon of central sensitization also resolved. This suggests that the identification of the original pain generator remains important in patients with a long history of chronic low back pain and that additional attention should be focused towards the thoracolumbar fascia, as full resolution of their pain complaint may still be possible.

## Conclusions

This case demonstrates that the identification of the primary pain generator should remain the top priority even in chronic back pain patients who have suffered for many years. This is the first case that not only documents a thoracolumbar fascial tear as a source of chronic back pain but also reports on successful treatment many years later. A search of the English literature in PubMed did not yield any articles on a specific diagnosis or treatment of the thoracolumbar fascia. It is important to consider the thoracolumbar fascia in the large differential diagnosis of chronic back pain and include ultrasound-guided blocks as a diagnostic modality and platelet-rich plasma as a potential treatment option.
